# Two Sides of Theory of Mind: Mental State Attribution to Moving Shapes in Paranoid Schizophrenia Is Independent of the Severity of Positive Symptoms

**DOI:** 10.3390/brainsci14050461

**Published:** 2024-05-02

**Authors:** Christina Fuchs, Sarita Silveira, Thomas Meindl, Richard Musil, Kim Laura Austerschmidt, Dirk W. Eilert, Norbert Müller, Hans-Jürgen Möller, Rolf Engel, Maximilian Reiser, Martin Driessen, Thomas Beblo, Kristina Hennig-Fast

**Affiliations:** 1 Department of Psychiatry and Psychotherapy, Ludwig-Maximilians-University, 80539 Munich, Germany; 2 Institute of Medcial Psychology, Ludwig-Maximilians-University, 80539 Munich, Germany; 3 Department of Clinical Radiology, Ludwig-Maximilians-University, 80539 Munich, Germany; 4 Department of Psychiatry and Psychotherapy, Universitätsklinikum OWL, 33617 Bielefeld, Germany; 5 Department of Psychology, Leopold-Franzens-University, A-6020 Innsbruck, Austria

**Keywords:** theory of mind, mentalizing, attribution, paranoid schizophrenia, acute and post-acute psychosis, functional magnetic resonance imaging

## Abstract

Background: Theory of Mind (ToM) impairment has repeatedly been found in paranoid schizophrenia. The current study aims at investigating whether this is related to a deficit in ToM (undermentalizing) or an increased ToM ability to hyperattribute others’ mental states (overmentalizing). Methods: Mental state attribution was examined in 24 patients diagnosed with schizophrenia (12 acute paranoid (APS) and 12 post-acute paranoid (PPS)) with regard to positive symptoms as well as matched healthy persons using a moving shapes paradigm. We used 3-T-functional magnetic resonance imaging (fMRI) to provide insights into the neural underpinnings of ToM due to attributional processes in different states of paranoid schizophrenia. Results: In the condition that makes demands on theory of mind skills (ToM condition), in patients with diagnosed schizophrenia less appropriate mental state descriptions have been used, and they attributed mental states less often to the moving shapes than healthy persons. On a neural level, patients suffering from schizophrenia exhibited within the ToM network hypoactivity in the medial prefrontal cortex (MPFC) and hyperactivity in the temporo-parietal junction (TPJ) as compared to the healthy sample. Conclusions: Our results indicate both undermentalizing and hypoactivity in the MPFC and increased overattribution related to hyperactivity in the TPJ in paranoid schizophrenia, providing new implications for understanding ToM in paranoid schizophrenia.

## 1. Introduction

The term theory of mind (ToM) describes the tendency to make inferences about other people’s mental states such as beliefs, desires, and goals [1,2]. These inferences are used to explain and predict the behavior of others and, thus, they are essential for successful social communication and interaction. Properly functioning ToM enables people to adopt new perspectives, to understand other people’s motivation to act, and to empathize with them [3]. 

However, ToM can be conceptualized in different ways. According to the approach on ToM as a mentalizing style [4], it can be described as the capability to make inferences about others’ mental states, thoughts, beliefs, and emotions, thereby interpreting, explaining, and predicting their behavior [5], Fonagy and Luyten [6] highlighted two processes involved in the socio-affective information process. The first (emotional mentalizing) refers to the automatic, implicit, or nonconscious and reflexive processing of external information about others (e.g., expressions, attitudes), while the second (cognitive mentalizing) refers to more explicit and voluntary levels of social–emotional processing. It is thought to be preferentially involved in the processing of information about others’ inner selves, such as their mental states and intentions. Moreover, the mentalizing model proposed by Fonagy and Luyten [6] argues that the ability to understand the behavior of others, in terms of their thoughts and feelings, is a developmental achievement. Thus, according to their approach, it is important to consider differences in the development of diseases, because unlike patients with autism spectrum disorders, patients with paranoid schizophrenia and positive symptoms would have to lose their representational understanding of the mind rather than never develop it, and, unlike patients with autism spectrum disorders, patients with positive-symptom schizophrenia would have to lose their understanding of theory of mind application rules rather than never develop them. Following this approach, it can be argued that theory of mind impairment, in terms of a complete lack of ToM abilities, does not apply to paranoid schizophrenia [4,7]. In line with this, a continuity model of ToM deficits has been suggested: (1) genuinely impaired ToM, (2) normal ToM without the ability to apply this knowledge, and (3) hyper ToM, associated with quantitative overgeneration of hypotheses or overattribution of mental states [4]. With regard to positive symptoms in schizophrenia and to the disorganized type of schizophrenia, they assume that patients do apply their knowledge others’ minds, but in an incorrect or biased way [4,8,9]. The transfer of one’s own knowledge to situational events can also be understood in terms of attribution theories, because these theories, similar to mentalizing approaches of ToM, aim to explain how human beings evaluate and determine the cause of other people’s behavior. While previous research has focused on deficits either in conceptualization (deficit or even lack of representational abilities) or in the application of these skills [8,10] (a non-social cognitive deficit), Abu-Akel’s hypertheory of mind [4] enables a differentiation between what patients theoretically do know of their own and others’ minds and what of their knowledge they practically apply in social settings. It also explains specificities in the theory of mind in schizophrenia like the cognitive attribution bias that reflects a predilection to use information about others’ mind in a particular way (i.e., overattributing knowledge to self and others). This cognitive bias might even be expressed in a hyperfunctional ToM, e.g., that individuals mentalize other people’s mental states in an exaggerated way [11,12]. This is termed overmentalizing or overattribution [13,14] in contrast to undermentalizing, which is characteristic of autism and refers to a deficit of ToM ability (i.e., reduced ability to understand and attribute mental states intentions), and complete lack of mentalizing (or no-mentalizing), i.e., entirely failing to attribute mental states [15,16]. Attribution focuses on an individual’s perception of the cause of events and behaviors.

### 1.1. Psychopathological Dysfunctions of ToM in Schizophrenia

Psychopathological abnormalities of ToM have thus far primarily been described in autism spectrum disorders [17], yet schizophrenia is also discussed to be characterized by ToM impairment, e.g., [18,19,20,21,22], which might be a key contributor to the poor social functioning in this disorder. In this regard, ToM deficits were confirmed in patients diagnosed with schizophrenia with predominantly negative symptoms, but these also already existed at the time of onset of the disease [11,12,23]. Even prior to the diagnosis of psychotic symptoms, deficits in social and emotional functioning are reported to be present [7,23]. Considerable evidence also indicates that other more basic cognitive impairments are also present in cases well before the onset of psychotic symptoms. Thus, socio-cognitive and negative symptoms are assumed to be reasonably characterized as “early symptoms”. They are discussed as being indicators of a developmental (e.g., premorbid to prodromal) component of schizophrenia. During the course of disease, even in cases where there is relative remission of psychotic symptoms, negative and cognitive symptoms are often found to be persistent. Thus, cognitive and negative symptoms appear to share a similar course [7,23]. Therefore, on the one hand, negative symptoms are assumed to be closely related to the severity of cognitive impairment. On the other hand, some findings also indicate a dissociation of both or at least a proportionate separation of both but probably based on the same neurobiological underpinnings [7,23].

Moreover, it was shown that individuals with pronounced disorganized symptoms [4,20] and thought disorders [24] have problems making mental attributions [22].

In contrast, with regard to positive symptoms, it is assumed that the proposed cognitive bias and overmentalizing [4] may underlie the difficulties in social attribution [25] and social judgement [26]. For example, schizophrenic patients with delusions have deficits in drawing appropriate conclusions from the evidence presented [4,9], but this concurs with previous findings suggesting that patients with positive-symptom schizophrenia do have a conceptual understanding of others’ minds but are not able to apply their knowledge in a correct way; thus, their faults are reflected in false conclusions about others’ mental states [9]. 

However, Scherzer et al. [21] stated that studying ToM in paranoid schizophrenia has led to a number of distinct ToMs due to different task demands. Their results indicate that ToM might be subdivided into separable dimensions: e.g., first- and second-order inferences or beliefs, interpretation of intentions, and interpretation of affect. Referring to ToM and cognitive capacities, Scherzer et al. [21] reported an independency of IQ on performing ToM demands. Contrarily, Sahl et al. [27] reported that global ToM impairment was negatively correlated with IQ. They concluded that intact higher-level reasoning may prevent the high-IQ group from making overmentalizing errors, through self-monitoring or inhibition. The authors proposed that high-IQ patients are chiefly impaired in lower-level ToM, whereas low-IQ patients also have impaired higher-level ToM. Conceivably, this specific impairment could help to explain the lower functioning reported in persons with intact IQ. However, in general, ToM impairments in patients suffering from schizophrenia have been associated with slower reaction times [28] and a decelerated cognitive processing speed as compared to healthy controls [19], whereas others assume no correlation of non-ToM cognitive capabilities and ToM functions [29,30,31]. Another approach to social cognition and ToM is the differentiation between the affective and cognitive dimensions of ToM [32,33]. Attributions of thoughts, knowledge, or action plans make up cognitive ToM, whereas attributions of emotional states like anger or guilt are referred to as affective ToM [34]. Significant positive relationships have been reported between cognitive ToM and positive symptoms, and between affective ToM and negative symptoms [30]. Contradictory findings on ToM impairment were also reported for the subtype of paranoid schizophrenia [35], as the authors only found a correlation between overmentalisation and positive symptoms. Although undermentalising was partially associated with disorganised symptoms, no correlation of ToM deficits with negative symptoms was found. The higher number of “reduced ToM” responses suggests that schizophrenia is characterized by accuracy problems rather than a fundamental lack of mental state concept.

### 1.2. Neurofunctional Findings of ToM in Healthy Samples and Samples with Schizophrenia

A growing number of functional brain imaging studies [36,37] indicate that as a higher cognitive function, ToM involves an expansive brain network. However, two brain regions seem to play a pivotal role: First, the medial prefrontal cortex (mPFC) [24,38], an area that is also activated while thinking about one’s own mental state [39] and during autobiographical memory retrieval [40]; second, the temporo-parietal junction (TPJ), which has been discussed as a ToM-specific brain region [37,41,42].

Some neuroimaging studies on ToM in schizophrenia reported hypoactivations [24,28,43] while others reported hyperactivations [41] within the ToM network, both demonstrating abnormalities particularly in the medial prefrontal network. For instance, when attributing intentions to acting comic figures in a ToM task, lower right prefrontal activation was detected in schizophrenia patients when compared to healthy participants [24]. The only study focusing on paranoid schizophrenia patients showed significantly less activity in the ToM network, particularly in the paracingulate cortex and bilateral TPJ [28]. Those aberrant activation levels were specifically related to tasks that require comprehension of social intentions, but not of non-social intentions.

### 1.3. The Moving Shapes Paradigm Adapted from Heider and Simmel (1944) and Its Neurofunctional Correlates

Within the broad spectrum of ToM, the perception of animacy, interactivity, and goal-directed behaviors derived from Heider–Simmel type animations [44] reflect the human tendency to construct social interpretations and derive inferences about beliefs and desires from movement patterns alone [45]. In a recent review, the authors [46] discussed the perceptual, developmental, and neural underpinnings of perceived animacy and social attributions. They provided support to link the development of neural systems to the ability to draw upon perceptual cues for animacy in order to establish more complex beliefs about the goals of others. At the turn of the century, Klin [47] developed a measure: the so-called social attribution task (SAT). Others, e.g., Abell et al. [48] and Castelli et al. [49] used comparable paradigms to investigate specific ToM deficits employing video sequences depicting two triangles as socially interacting geometric moving shapes, both tasks adapted from the original paradigm by Heider and Simmel [44]. This classic social mental attribution paradigm using animated shapes as stimuli is a relatively underutilized method of modeling social interactions and mental state attributions. It relies on individuals’ ability to make social inferences and judgments from geometric animated stimuli, and it is a convenient way to elicit social attribution while avoiding some of the limitations of other methods. Not only do these animated tasks typically require less reading or verbal ability, but they have been reported to display little to no cross-cultural difference [50]. Today’s research has confirmed the spontaneous attribution of social meaning to the videos. The video material was reported recently to be a valid and reliable measure of social attribution by varying how many social attributions are made in response, and the videos varied in how much they elicited such responses [51,52]. The paradigm correlates positively with measures of adaptive functioning [53] and other ToM tasks [54]. The paradigm had already been adapted to the imaging environment and it has been shown to activate the neuronal ToM network [49].

The relevant neuronal networks that are functionally involved in social cognition to perceived animacy from animations of simple shapes have been investigated by several prior fMRI and PET (positron-emission tomography) studies: Using fMRI, Gobbini et al. [36] investigated the neural responses of human adults to animations involving rigid social interactions that conveyed goal-directed action and to false-belief stories. Interestingly, and consistent with previously reviewed behavioral reports, two distinct systems were evoked by goal-directed animations and mentalistic stories. These systems were widely distributed, but notably involved the posterior superior temporal sulcus (pSTS) for representations of goals and the temporo-parietal junction (TPJ) for mental state attributions, areas known as part of the neural system for theory of mind. Using PET, both the pSTS and the TPJ were also found to be involved when watching Heider–Simmel-like shape animations in a ToM condition [49]. The authors also reported the involvement of the medial prefrontal cortex (mPFC), a midline structure associated with introspective thought, when viewing ToM animations. Martin and Weisberg [55] found evidence that ToM animations in a moving shape paradigm engage the “social brain network” (patterns of neural activity bilaterally on the STS and within ventral parts of the mPFC (vmPFC)). Since the identified regions are also part of the default mode network (DMN) in adults [46], both systems can be assumed as overlapping.

### 1.4. Study Aims

The question remains as to whether deficits of ToM in paranoid schizophrenia manifest as a reduced ability of introspection and mentalizing, reflected by a flattened ToM network activation, or rather as a tendency to make faulty, delusionally overreaching attributions of others’ mental states, associated with hyperactivation of the ToM network [15], and how these tendencies are associated with negative and positive symptomsSeveral studies indicate that ToM deficits decrease in the remission phase [9,56,57]. It is therefore also being discussed whether this occurs in parallel with the decrease in cognitive dysfunction during remission, contrary to earlier assumptions of persistent cognitive impairment even during remission [21]. In accordance, studies provided support for ToM impairments as stable during the course of disease from an acute state to remission [20,58,59].

To overcome the still-conflicting findings of ToM and its underlying neurobiological mechanisms, we investigated patients with different states of development of disease differentiating with regard to positive symptoms. Twenty-four acute (APS) and post-acute (PPS) patients with diagnosed paranoid schizophrenia were examined with the adapted Heider and Simmel moving shapes paradigm at the behavioral level but also at the neurofunctional level. Comparable variations of this dynamic intentional movement interpretation task have previously been used in prior research (e.g., [49,60]) compared to healthy controls. 

For our study, we assume the following:

(a) Both patient groups attribute fewer intentions to the moving figures in the experimental task and recognize the mental states of the acting figures less (“undermentalizing”) than healthy individuals, as the deficit is presumably more associated with negative symptoms, which are more state-independent than positive symptoms. This can be shown using the behavioral findings of the experimental ToM condition.
(b) The acute patient group differs in the attribution of intentions to figures from the post-acute patient group and healthy participants, as the over-interpretation of mental states of the moving figures (“overmentalizing”) is presumably more related to the positive symptoms, which should therefore differ between the states of the illness, which can be shown using the behavioral findings of the experimental random condition.

With regard to underlying neurofunctional mechanisms we hypothesize the following:

(a) The neural correlates of “undermentalizing” in the ToM condition are reflected by reduced brain activity in the ToM network in both patient groups when being compared to the healthy group.
  We also assume the following:
(b) The correlates of “overmentalizing” in the random condition are reflected by increased brain activity in the ToM network especially in the patient group with acute positive symptoms.

## 2. Methods

### 2.1. Participants

Thirty-six native-speaking German participants aged from 21 to 59 were included in the study. Twenty-four participants met the ICD-10 (International Classification of Diseases) [61] criteria for paranoid schizophrenia. Symptom specificity was rated by the attending doctors using the German version of the Structured Clinical Interview for DSM IV (SCID, Axis I and II) [62] and the Positive and Negative Symptom Scale (PANSS) [63]. Since acute paranoid schizophrenia is characterized by a distinctive positive symptomatology, the positive scale of PANSS was used to discern 12 acute (APS) and 12 post-acute (PPS) patients with the diagnosis of psychotic schizophrenia (cut-off value 14, calculated with a median split on the positive scale of all patients with diagnosed schizophrenia). Thus, APS patients, regardless of the number of acute psychotic states before, were defined by an acute state of psychosis and a pattern of positive symptoms. In contrast, PPS patients exhibit fewer positive symptoms and were recruited in a state at a maximum of 6 months after the acute exacerbation of psychosis (the calculated group differences in the PANSS can be found in the Results Section, Table 2). Both groups with diagnosed schizophrenia received parallel medication with atypical neuroleptics. Twelve demographically matched healthy individuals with no history of relevant medical, psychiatric, or neurological illness were recruited via flyer advertisement and investigated regarding healthiness using the SCID [62]. The three groups each consisted of eight male and four female participants and did not significantly differ with respect to age (F(11, 2) = 0.93, *p* = 0.403), premorbid verbal intelligence (F(11, 2) = 0.13, *p* = 0.875), education (F(11, 2) = 1.35, *p* = 0.178), and handedness (F(11, 2) = 1.41, *p* = 0.197). To control for the effect of pharmacological treatment on information processing, an alertness test (TAP “Alertness”) [64] was conducted using reaction times as a critical parameter. No differences were found between the three experimental groups (F(11, 2) = 1.56, *p* = 0.224, see Table 1).

#### Inclusion and Exclusion Criteria

To ensure that all study participants were able to fulfil the requirements of the experimental task, a language-based intelligence test was completed. We aimed to measure ToM skills independent of intelligence rather than intelligence-related performance in the experimental investigation. Thus, only participants with an intelligence quotient of at least 85 were included in the study. IQ was measured using a language-based intelligence test (German: WST—Wortschatztest [65]), which enables an assessment of the age- and developmentally stable verbal intelligence level and an evaluation of language comprehension. Furthermore, the general inclusion criteria for all participants included an age of 20 to a maximum of 60 years and German mother tongue for all participants and, for the patient groups, a medically confirmed diagnosis of paranoid schizophrenia (F20.0) in an acute or post-psychotic state according to ICD-10. 

Suspected or prior brain damage (e.g., traumatic brain injury or meningitis in the past), neurological diseases, or other co-morbid initial diagnoses of Axis-I diseases (also in family history), current or prior substance abuse, and acute suicidal behavior were exclusion criteria. Other exclusion criteria relate to the fMRI examination, all subjects were also excluded if they had metal parts such as a pacemaker in their body, were claustrophobic, and if the female participants were pregnant, which is why a total of 6 people had to be excluded before the study.

The healthy volunteers were recruited on the one hand by personally approaching their circle of acquaintances and on the other hand by flyers. In order to recruit patients diagnosed with paranoid schizophrenia, flyers were distributed after a detailed presentation of the study to the psychiatrists and psychologists on all wards where schizophrenic patients were treated, the day clinic, and the outpatient clinic of the Clinic for Psychiatry and Psychotherapy at the LMU in Munich.

The study received approval from the local research ethics committee of the Medical Faculty of LMU Munich and is in accordance with the Declaration of Helsinki and subsequent revisions. Written informed consent was obtained from all participants.

### 2.2. Stimulus Material

**Figure 1:**
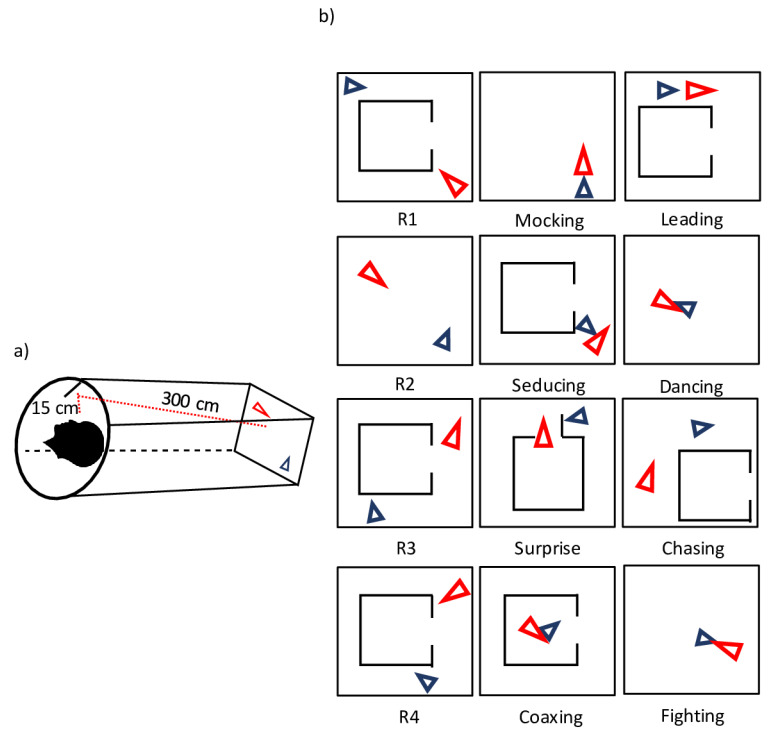
(**a**) Participant in fMRI scanner and mirrored video clip. (**b**) The three experimental conditions with 12 video clips adapted from Heider and Simmel [44]. The different colours of the arrowheads were used to make it easier to visually distinguish between the two moving shapes.

The moving shapes paradigm for exploring mentalizing used in this study was adapted from Castelli and colleagues [49] and is based on the study of Heider and Simmel [44], who demonstrated that simple geometrical shapes can evoke the attribution of intentions, being perceived as acting persons rather than abstract figures when moving in a particular way. The animated sequences comprised four “random” (R), four “goal-directed” (GD), and four “ToM” animations, lasting between 34 and 45 s. All of them presented a big red triangle and a smaller blue one, moving on a white screen. In the random condition (R), which can be taken as a visuo-perceptive baseline, triangles move indiscriminately, as if they are floating or bouncing. For the goal-directed (GD) condition, which represents an intermediate level between the R and ToM condition, an understanding of simple intention is required. The triangles interact in a purposeful way, for example, chasing one another, whereas, in the ToM condition, the triangles interact in socially complex ways, containing actions and reactions of the two triangles, implying an understanding of “minds”. While the type of movement was by definition different between the three conditions, the basic visual characteristics in terms of shape, overall speed, and orientation changes were as similar as possible (see Figure 1). The requirement of spatial and temporal awareness when assessing ToM makes this paradigm powerful in measuring real-world demands. Hence, our paradigm seems to be an appropriate method for investigating whether paranoid schizophrenia patients show—on the level of behavior and brain activity—undermentalizing or overmentalizing. Possible differences between APS and PPS may answer the question of ToM being a state or trait marker.

### 2.3. Experimental Procedure

**Figure 2:**
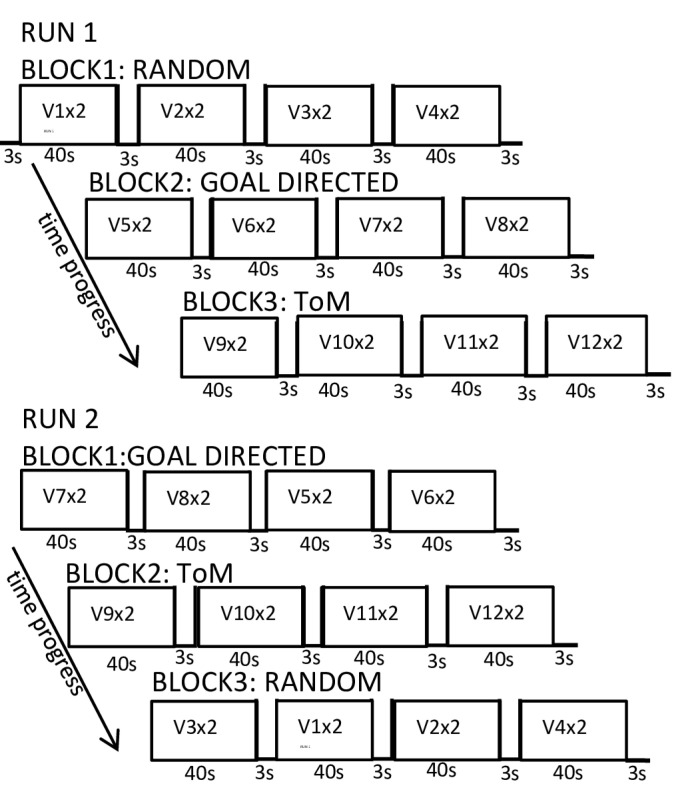
fMRI: Experimental block design: Two runs with three blocks each (random, goal-directed, theory of mind conditions). Each block consists of 4 video clips of one condition, each of which repeated twice.

The single animations [49] were modified for fMRI (Presentation 0.80, Neurobehavioural Systems; http://www.neurobs.com/) and shown for a duration of 20 s. Participants were instructed to partake in a study on the perception of movement, to watch short animated film sequences, and think about what was happening. After ensuring the task was understood by the participant, all of the 12 animations were presented twice (to obtain an intensification of effects) in two runs in a block design (R, GD, ToM) using a pseudo-randomized order within each block and between blocks. Each run began with a three-second presentation of a white screen, followed by the three blocks with four animations, respectively, and inter-stimulus intervals of three seconds (see Figure 2). In total, the experimental task lasted about 20 min. After scanning, each participant watched the animations once again, presented on a computer in the same order and size as presented before. They were asked about their thoughts and impressions of the different conditions in open questions. No detailed feedback was given for these observations, apart from general positive comments.

### 2.4. Scoring and Evaluation

The verbal descriptions given after each presentation were coded along three dimensions and rated on Likert scales. “Intentionality”, i.e., the degree of attributing a mental state (0 = non-deliberate action to 5 = deliberate action aimed at affecting another’s mental state) and “appropriateness”, i.e., how well the underlying script of the presented actions was captured (0 = inappropriate or no answer to 3 = appropriate and clear answer) were adapted from Castelli et al. [49]. Following the idea of Heider et al. [44], the dimension “humanization” was added to survey the degree of humanization of the acting figures (0 = speaking of triangles without any humanization to 3 = speaking of human beings). The complete codes can be seen in Appendix A.

To test inter-rater reliability, the three raters’ coding consistency with respect to the participants’ answers was calculated by Fleiss’ Kappa. Since all values were between 0 and 1 throughout all three conditions and dimensions (RAppropriateness = 0.53, RIntentionality = 0.57, RHumanization = 0.60, GDAppropriateness = 0.44, GDIntentionality = 0.31, GDHumanization = 0.59, ToMAppropriateness = 0.50, ToMIntentionality = 0.38, ToMHumanization = 0.76), we proceed with the assumption of a medium inter-rater reliability that reflects the appropriateness and validity of the chosen stimuli. 

### 2.5. Data Acquisition

Stimuli were presented using a stimulus delivery software (Presentation 0.80, Neurobehavioural Systems). They were projected onto a translucent screen by a commercially available video beamer (INTouch, resolution of 1024 × 768 pixel). Participants viewed the stimuli over a head-coil compatible mirror system (300 cm screen to mirror, 15 cm mirror to participant’s eyes, see Figure 1).

MRI imaging was performed at a 3.0T field strength (Magnetom Verio, Siemens, Erlangen, Germany) using a T2*-weighted echo planar imaging (EPI) sequence with the following parameters: repetition time (TR) = 3000 ms, echo time (TE) = 30 ms, flip angle (FA) = 90°, matrix size = 256 × 230, field of view (FoV) = 256 mm, pixel size = 3 mm × 3 mm, slice thickness = 3 mm). The axial images were oriented parallel to the anterior commissure–posterior commissure (AC-PC), specified with a midsagittal scout image. Thirty-six transversal slices from the cerebellum to the cortex were acquired in interleaved order. A respective functional experiment consisted of 96 volumes. Two functional runs were acquired in total. In order to avoid a T2 saturation effect, we did not present any material during the first four volumes and excluded the first three volumes from further analyses. 

For anatomical reference, high-resolution anatomical images (MPRAGE) were recorded (TR = 3000 ms, TE = 30 ms, flip angle = 90°, FOV = 256 mm, matrix size = 256 × 230, 160 sagittal slices, in-plane resolution 1.05 mm × 1.05 mm, slice thickness = 1.25 mm).

### 2.6. Statistical Analysis

Behavioral data were analyzed using the Statistical Package for the Social Sciences (SPSS 17.0). Significance levels were defined as *p* < 0.05. All data were corrected for multiple comparisons with the Bonferroni procedure and a significance level of *p* < 0.05. A multivariate analysis of variance (ANOVA) and several Chi-square tests were used to calculate differences between all three groups.

Analyses of the neuroimaging data were performed using BrainVoyager QX software [66]. In preprocessing the data, all images were corrected for motion and slice-scan time order, temporally and spatially smoothed, mean-intensity-corrected, co-registered with the participants’ corresponding anatomical (T1-weighted) images, and transformed to a Talairach standard coordinate system. After data preprocessing, a random effects general linear model (GLM) with predictors for all three conditions (R, GD, ToM) was computed. In a voxel-based approach, contrast maps were created for the three conditions within each of the participant samples. Standard stereo-tactic coordinates for the voxel displaying local maximum activation were determined within the areas where significant relative changes in neural activity were found. These local maxima were anatomically localized by reference to a standard stereo tactic atlas [67] using TalairachClient (2.4.2).

On the second level of statistical analysis, we performed a two-factorial model using the group (APS, PPS, controls) and experimental conditions (ToM, R) as factors (3 × 2). A random effects analysis was calculated to deduce the overall characteristics across different individuals. To investigate differences between acute and post-acute schizophrenia patients, as well as between patient groups and healthy participants, both within-subject and between-subject contrasts were calculated. In our statistical model, R was used as a control condition, and activation levels during the ToM condition were calculated as compared to this baseline. In addition, to detect overlaps in activation patterns over all three groups during ToM tasks, a conjunction analysis was performed.

## 3. Results

As our study investigates ToM abilities in schizophrenia, our reported findings focus only on a comparison between the ToM condition and the random condition as a visuo-perceptive baseline.

### 3.1. Clinical Differentiation of Patient Groups

Both patient groups, acute and post-acute schizophrenia, differed significantly regarding positive symptoms, in particular regarding delusions, but did not differ regarding negative symptoms (see Table 2).

### 3.2. Behavioral Experimental Data

For the random animations, there was a significant difference between APS, PPS, and healthy controls in appropriateness, with a tendency towards less appropriate descriptions in the patient samples (“appropriateness”; F(2) = 3.66, *p* = 0.037; post-hoc Bonferroni correction: pAPS = 0.099, pPPS = 0.060), reflecting that patients did not perceive the movements as random. No significant differences between the three groups could be found in the perception of intentional movements (“intentionality”; F(2) = 1.59, *p* = 0.218) and in humanizing of the geometrical figures (“humanization”; F(2) = 1.34, *p* = 0.275), both attribution styles were seldom seen in the random condition. For the goal-directed condition, we found a significant difference solely between patients in the acute state and healthy participants regarding the appropriateness (F(2) = 5.32, *p* = 0.009).

For the ToM animations, the results revealed that APS as well as PPS used significantly less appropriate mental state descriptions (“appropriateness”; F(2) = 12.55, *p* < 0.001; post-hoc Bonferroni correction: pAPS < 0.001, pPPS = 0.001) than control participants. Furthermore, both groups with diagnosed schizophrenia significantly less often attributed mental states to the figures (“intentionality”; F(2) = 6.69, *p* = 0.004; post-hoc Bonferroni correction: pAPS = 0.013, pPPS = 0.008) than controls. While there was no difference in humanization of the moving figures between all three groups, there was a marked trend that APS evaluated the figures as being less human compared to healthy participants (“humanization”; F(2) = 2.99, *p* = 0.064; post-hoc Bonferroni correction: pAPS = 0.064 (see all differences in Table 3)).

### 3.3. Neuroimaging Data

Taking the findings of Pedersen and colleagues [19] into account, we controlled for temporal changes by adding a temporal comparison between the first and the second half of the animations. In contrast to previous findings, we could not find any significant difference in processing ToM-related tasks at different time points in any of our groups (all *p* > 0.05) and, hence, we do not provide further data description of this aspect. 

#### 3.3.1. Main Effect and Interaction Analysis of Group (APS, PPS, Controls) and Animation (ToM versus Random)

A main effect of the group was shown in the frontal (inferior frontal and precentral gyrus) and limbic (parahippocampal gyrus) regions, as well as in the TPJ and primarily in the occipital (inferior occipital gyrus) regions. A similar pattern of activations was found for the main effect of the animation condition in the frontal (medial and inferior frontal gyrus), limbic (cingulate gyrus), and temporal (fusiform and superior temporal gyrus), as well as in the occipital (fusiform, lingual, and inferior occipital temporal gyrus) regions. An interaction of the group and animation conditions could be related to differential activation levels in the TPJ.

#### 3.3.2. Within-Subject Random Effects Analysis of ToM Compared to the Random Condition

Within-subject contrasts in healthy participants revealed that the ToM condition was associated with increased activations in a large neuronal network, particularly in the prefrontal (superior, medial, and inferior frontal gyrus), limbic (uncus, parahippocampal gyrus, posterior cingulate cortex), temporal (medial and superior temporal gyrus), and occipital (medial and inferior occipital gyrus) brain regions, as well as in the TPJ as compared to the random condition. In APS, increased neural activation in the ToM condition was found in the frontal (medial prefrontal gyrus), limbic (posterior cingulate), and parietal (precuneus) brain regions and in the TPJ compared to the random condition. Increased neural responses associated with ToM compared to the random condition in PPS were located in the temporal (superior and inferior temporal gyrus), parietal (supramarginal and postcentral gyrus, inferior parietal lobule), and occipital regions (medial and inferior occipital gyrus, fusiform gyrus) and in the TPJ, as well as in small clusters in the prefrontal (inferior frontal gyrus) and limbic (parahippocampal gyrus) parts of the cortex.

**Figure 3:**
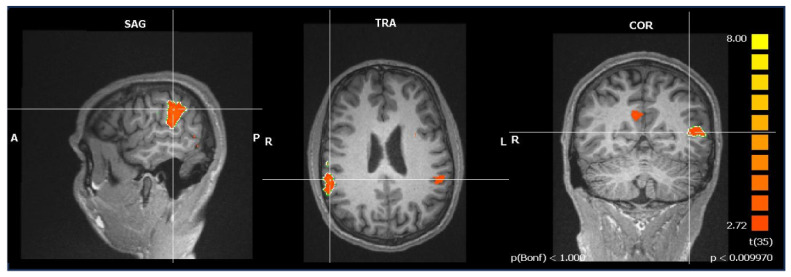
Activation of the right temporo-parietal junction while watching ToM > random animations as observed in all three samples; figure depicts exemplarily the activation pattern in post-acute patients with psychotic schizophrenia (Talairach coordinates 57, −37, 28; *p* < 0.001; Bonferroni-corrected); statistical pictures show the activation on a color scale from red to yellow, with yellow symbolizing stronger activation and red symbolizing weaker activation.

In summary, only a distinctive frontal activation was found in healthy participants that was not as prevalent in APS and PPS; whereas, in all three samples, ToM-specific activation in the TPJ could be found (see Figure 3, Table 4). No higher activation levels could be found for the inverse comparison of the random compared to the ToM condition.

#### 3.3.3. Between-Subject Random Effects Analysis of ToM Compared to the Random Condition

**Figure 4:**
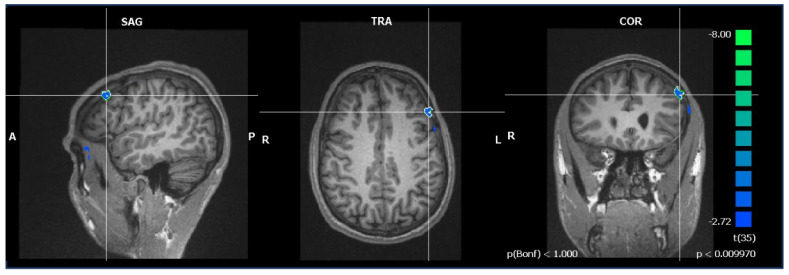
Decreased neural activation within the left medial prefrontal cortex while watching ToM > random animations in both patient groups compared to healthy controls; figure depicts exemplarily activation pattern in healthy controls compared to patients with acute paranoid schizophrenia (Talairach coordinates −48, 26, 40; *p* < 0.01; Bonferroni-corrected); statistical pictures show the hypoactivation on a color scale from blue to light green, with light green symbolizing stronger hypoactivation and blue symbolizing weaker hypoactivation.

Between-subject contrasts (see Figure 4, Table 5) of healthy controls compared to APS in the ToM condition compared to the random condition revealed higher activation levels in the prefrontal (superior frontal gyrus, medial prefrontal gyrus, inferior frontal gyrus), limbic (uncus, parahippocampal gyrus, posterior cingulate cortex), and sublobal (lentiform nucleus, putamen) regions, as well as in the TPJ. Compared to PPS, healthy participants demonstrated higher activations in the prefrontal (medial prefrontal gyrus), limbic (cingulate gyrus), and sublobal (caudate nucleus, lentiform nucleus) regions.

The inverse comparisons revealed only few brain regions with higher activations for APS or PPS, respectively, compared to healthy controls: In APS, those were located in the prefrontal (medial prefrontal gyrus) and limbic (parahippocampal gyrus, cingulate gyrus) regions, while in PPS they were located in the cingulate gyrus.

## 4. Discussion

Our current study aims at a deeper understanding of ToM impairments based on the Heider and Simmel moving shapes paradigm in paranoid schizophrenia, in particular whether they are caused by undermentalizing or by overmentalizing. Our findings indicate two sides of ToM in paranoid schizophrenia that might be impaired separately. 

On a behavioral level, patients suffering from schizophrenia both in an acute as well as a post-acute paranoid state interpreted the animations in the random condition as less random than healthy participants. Additionally, they gave fewer and less accurate interpretations of animations in the ToM condition, reflected in reduced appropriateness and in heightened intentionality attributed to others. These findings are consistent with a number of previous studies that found clear behavioral ToM deficits in persons suffering from paranoid schizophrenia and first-episode patients with schizophrenia, e.g., Harrington et al. and Koelkebeck et al. [59,60], and can be understand as overmentalizing that is independent from the state acuteness of illness with respect to positive symptoms. Moreover, the group of APS in our study tended to humanize the geometric figures less than healthy individuals in the ToM-related task, indicating a perceptual bias and inferential bias especially in ambiguous social interactions in an acute state of paranoid schizophrenia, which can also be interpreted as undermentalizing. This was already proposed by Blackwood et al. [13] and McCabe et al. [14]. 

The healthy formation of social beliefs depends on the selection of social environmental data (attentional and perceptual bias) and on inferential processing using these data (inferential bias) as discussed by Abu-Akel et al. [4] in their approach of hypermentalization. The authors refer to a cognitive bias in schizophrenia that might explain both over- and undermentalizing. Their assumptions render the dichotomous classic tests of theory of mind (presence or absence of mentalization) inadequate in distinguishing between the reasons why people fail to answer belief questions accurately and rather indicate a continuous ability to mentalize. 

**Figure 5:**
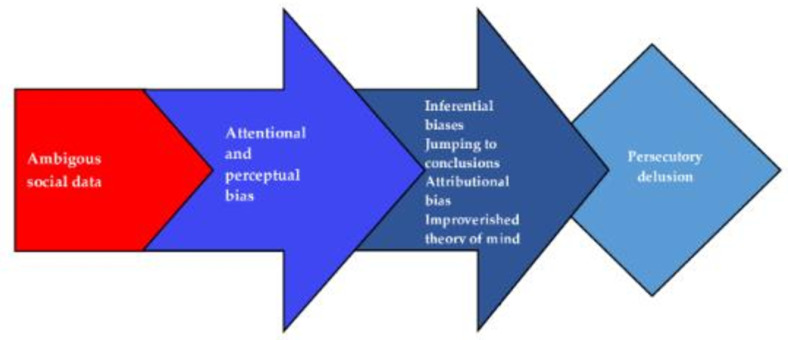
Biases in social belief formation probably support the development and maintenance of persecutory delusions (adapted from Blackwood et al. [13]).

Regarding the clinical impact of this mentalizing bias, it has also been proposed to have an etiological impact in the formation and maintenance of persecutory delusion (see Figure 5).

On a neurofunctional level, we found differential processing correlates with respect to (a) groups and (b) animation conditions. The differences comprise a network of frontal, limbic, and occipital brain regions, as well as the TPJ. 

In the group comparison, as a priori assumed, healthy controls exhibited increased activation within the ToM brain network compared to both APS and PPS. In particular, engagement of prefrontal regions including the MPFC and the superior prefrontal gyrus points to cognitive processes related to ToM and self-awareness [24,38,68]. This distinctive hypofrontality (mainly of the MPFC) during a ToM task in APS and PPS in comparison to healthy controls is consistent with previous findings [18,24]. Our results also substantiate an association of MPFC activation and ToM capabilities [24,38]. Together with our behavioral findings, the hypoactivation of MPFC suggests undermentalizing in paranoid schizophrenia and a probable dysfunction in anterior regions within the default mode network.

Group differences in activation levels of parts of the limbic system (e.g., the parahippocampal gyrus and uncus) that are relevant for memory processes might indicate the role of autobiographical memory retrieval in ToM. Supporting this notion, involvement of the parahippocampal gyrus has previously been found to contribute to the processing of social scenarios as complex visual stimuli. The activation of the posterior cingulum can be explained by prior findings suggesting its responsiveness in “false belief” tasks when participants know about other people’s beliefs [36].

Given that participants with paranoid schizophrenia were impaired in making correct mental state attributions, one might expect to find a general reduction in ToM-related neural activity in individuals suffering from paranoid schizophrenia [18,28], including not only less activity in prefrontal regions, but also in the TPJ. In contrast, we found significantly less TPJ activation only for APS, not for PPS, as compared to healthy individuals. This is in accordance with our findings on the behavioral level demonstrating less humanization and thus less reference to human social interactions during the ToM task in APS.

At the same time, a general involvement of TPJ while watching ToM animations compared to a control condition occurred in all three samples. This implies that cognitive processes related to ToM and attributing mental states to others are also prevalent in schizophrenia, but probably more biased in APS. In light of a “dynamic ToM network”, our results indicate that the posterior region TPJ might be involved in a pre-stage of ToM processing, e.g., in emotional perception and attribution [69] or self–other distinction. As the TPJ is not selective to processes related to social cognition [42], disentangling its functional role within the ToM network remains an aim for future research endeavors.

Furthermore, our findings support the notion of ToM deficits being only in some part related to positive symptom severity but rather to negative symptoms because both patient samples, APS and PPS, showed ToM-related alterations. Thus, on the one hand ToM impairment can be in some part considered a stable marker in schizophrenia, which is congruent with a number of previous findings. For example, Russell and colleagues [70] reported a significantly poorer description of moving geometrical objects in the ToM condition in all subtypes and phases of schizophrenia patients (including remitted ones). A further indicator for ToM deficits even as a trait marker is that ToM demands are also impaired in clinically healthy first-degree relatives of schizophrenia patients, but not in genetically clear (unrelated) participants [71]. Nevertheless, for answering the question of state or trait more precisely, a longitudinal study design also including subjects at risk of psychosis and at transition to psychosis is more appropriate, as proposed by Harvey et al. [7]. One study in children who developed schizophrenia spectrum disorders in adulthood already showed perspective-taking deficits in early infancy [72], substantiating the hypothesis that a ToM impairment may be a trait marker of schizophrenia based on possible early life-time changes probably related to later dysfunctional neural development within the MPFC and the anterior DMN [46,73]. But, on the other hand, ToM capabilities seem to also be influenced by pathological states in schizophrenia [35,74,75]. Accordingly, we found a posterior ToM-related brain region, the TPJ, only less responsive in the acute paranoid phase accompanied by less humanization in this group on the behavioral level. If our results are also considered in light of the findings on processing time in ToM-related tasks in schizophrenia [19], our data could be explained by slowed processing of ToM-relevant stimuli or alternatively due to disorganised thinking [74,75]. In our study, we tested it by adding temporal comparisons of activation patterns, it did not reveal any significant time processing difference in ToM-related tasks in our data and we even found non-deviant reaction times during an alertness task in both patient groups.

Nevertheless, for answering the question of state versus trait of these specific ToM deficits more precisely and the correlation with other cognitive functions, a longitudinal study design is more appropriate [7]. A study in children who developed schizophrenia spectrum disorders in adulthood showed perspective-taking deficits in early infancy [72], substantiating the hypothesis that a ToM impairment may be a trait marker of schizophrenia probably based on changes prior adolescence and first diagnosis. Further, recent evidence was provided by Thomas, Ryan and Gilman [76] that resting state network (DMN) is associated with cognitive flexibility during adolescence. Hence, we might assume changes in the DMN and social brain network probably caused by fetal or early infant disruptions of brain development to underlie the later observed social and cognitive deficits in adult schizophrenia. The longitudinal comparison pre-clinical subjects at risk of psychosis, prodromal psychosis, first-episode psychosis with no-drug intake, chronic psychosis and different patient groups with different classification of schizophrenia might help to identify the correlation of clinical symptoms, course of disease, drug intake and socio-cognitive performance as well as the correlation with underlying changes in neural connectivity as proposed by Guo, et al. [77].

### Limitations

Several limitations should be addressed when interpreting the present results. First, the present study focuses only on neural brain activity but not on connectivity. It enhances the specificity of the findings only for several brain regions but not for connective networks like the DMN. Second, the sample size is small. A large sample size is needed to elucidate the subtle changes in brain activity with regard to this specific task’s demands in schizophrenia. Finally, we did not focus on first-episode, drug-naive patients with paranoid schizophrenia; hence, our findings might be related to drug intake and the course of schizophrenia. However, our results are largely consistent with previous studies in this field.

Instead of family-based case–control, we used traditional case–control designs. In future, more family-based case–control designed studies could limit the confounding effects of environmental factors in our comparisons of participants suffering from schizophrenia and heathy participants.

Despite the limitations, the present study observes hypoactivity and hyperactivity within the social brain network in patients with paranoid schizophrenia related to undermentalizing and overattribution. Our findings support the need for further studies on the neurofunctional level to provide a better understanding of symptom-related changes in “theory of mind” functions and to better understand differences between subtypes of schizophrenia. Still, conceptual work on measures of investigating ToM is needed as also proposed by others [30,35,74,75].

## 5. Conclusions

In conclusion, ToM deficits in schizophrenia can be linked to dysfunctional MPFC activation in acute and post-acute psychotic patients indicating undermentalizing as a stable trait marker independent of the status of disease. At the same time, increased over-attribution to others was found. On a neural level, two dissociated network functions seem to contribute to ToM impairments in paranoid schizophrenia, i.e., constant hypoactivation in the MPFC and variable aberrant activation in the TPJ. This functional dissociation inheres relevant implications for an understanding and modeling of ToM abilities as assumed by [4] in their hypertheory of ToM. A stable pattern of brain activation in paranoid schizophrenia patients, which is independent of the severity of positive symptomatology, provides novel insights to inform the debate on ToM deficits as state versus trait markers in schizophrenia. By invoking the notion of hyper-theory of mind, impairment in mental representation in paranoid schizophrenia may be understood as being located on a continuum. It is for future research to define methods to capture this proposed continuum of theory of mind impairments in longitudinal studies from prodromal to chronic schizophrenia.

## Figures and Tables

**Table 1:** Demographic description of samples.

Patients with Diagnosed Schizophrenia					Healthy Participants		
	Acute		Post-Acute				
	(n = 12)		(n = 12)		(n = 12)		
	M	SD	M	SD	M	SD	*p*
Age (range: 21–59 years)	35.7	10.14	39.58	12.99	37.75	8.17	0.403
IQ	105.17	11.56	105.92	12.80	107.58	10.65	0.875
Education (range: 9–14 years of education)	1.91	0.67	2.33	0.89	2.25	0.87	0.178
Alertness Raw values of reaction times	263.25	91.7	266.58	59.73	223.83	32.05	0.224

Legend: M = mean, SD = standard deviation, *p* = significance (2-tailed), α = 0.05, equal variances were assumed.

**Table 2:** PANSS scales (positive, negative, general, and sum scale) in acute and post-acute patients with paranoid schizophrenia.

Patients with Diagnosed Paranoid Schizophrenia					
	Acute		Post-Acute		
	(n = 12)		(n = 12)		
	M	SD	M	SD	*p*
PANSS pos	17.83	4.45	10.75	2.09	0.000
PANSS neg	18.83	5.86	19.17	7.51	0.905
PANSS gen	40.83	11.35	33.0	7.25	0.178
PANSS total sum score	77.50	20.05	62.92	15.23	0.224

Legend: M = mean, SD = standard deviation, *p* = significance (2-tailed), α = 0.05, equal variances were assumed. Abbreviations: PANSS pos = positive symptom scale; PANSS neg = negative symptom scale; PANSS gen = PANSS general psychopathological scale; PANSS total sum score = sum score of all PANSS scales.

**Table 3:** Group differences in ratings of the moving shapes paradigm video clips.

Patients with Diagnosed Schizophrenia					Healthy Participants		
	Acute		Post-Acute				
	(*n* = 12)		(*n* = 12)		(*n* = 12)		
	M	SD	M	SD	M	SD	*p*
Random A	1.95 1.95	0.62 0.62	1.90 1.90	0.55 0.55	2.45 2.45	0.46 0.46	1.000 0.099 0.060
Random I	0.45 0.45	0.51 0.51	0.84 0.84	0.61 0.61	0.52 0.52	0.54 0.54	0.309 1.000 0.537
Random H	0.67 0.67	0.81 0.81	0.71 0.71	0.72 0.72	0.31 0.31	0.28 0.28	1.000 0.574 0.436
GD-A	2.09 2.09	0.51 0.51	2.29 2.29	0.48 0.48	2.65 2.65	0.23 0.23	0.739 0.009 0.147
GD-I	2.50 2.50	0.54 0.54	2.28 2.28	0.31 0.31	2.40 2.40	0.36 0.36	0.614 1.000 1.000
GD-H	1.93 1.93	0.80 0.80	1.46 1.46	0.77 0.77	1.73 1.73	0.47 0.47	0.338 1.000 1.000
ToM—A	1.70 1.70	0.62 0.62	1.75 1.75	0.60 0.60	2.63 2.63	0.20 0.20	1.000 0.000 0.001
ToM—I	2.91 2.91	1.25 1.25	2.83 2.83	0.96 0.96	4.09 4.09	0.38 0.38	1.000 0.013 0.008
ToM—H	1.73 1.73	0.79 0.79	1.98 1.98	0.78 0.78	2.41 2.41	0.42 0.42	1.000 0.064 0.406

M = mean, SD = standard deviation, *p* = significance (2-tailed), α = 0.05, equal variances were assumed. GD = goal-directed, ToM = theory of mind, A = appropriateness, 0–3, from “no answer“ (0) to “clear, appropriate answer” (3), I = intentionality = degree of attribution of mental states, 0–5, from 0 = “undeliberate action” to 5 = deliberate action aimed at affecting another’s mental state, both adapted from Castelli, Frith, Happé, and Frith [49], H = humanization = humanizing description of the figures, 0–3, from “no human description” (0) to “speaking of human beings” (3), self-nominated category (for more details see Appendix A).

**Table 4:** Neuronal activation ToM–Random in each investigated group (within-contrast) and vice versa (Random–ToM).

Talairach Coordinates							
Brain Region	BA	x	y	z	Z	r/l	Voxel
***Theory of Mind > Random***							
**Acute Patients with Paranoid Schizophrenia**							
**Lobus Frontalis**							
Gyrus frontalis medialis	8	−12	38	37	3.58	L	292
**Lobus limbicus**							
Cingulum posterior	31	6	−55	25	3.41	R	462
**Lobus temporalis**							
Gyrus temporalis superior	22	48	−16	−5	3.40	R	269
Gyrus temporalis superior	41	42	−34	7	3.26	R	101
Gyrus temporalis medialis	21	48	−31	−2	3.47	R	339
Gyrus temporalis medialis	39	−45	−55	7	3.48	L	1187
Gyrus fusiformis	37	−45	−40	−2	3.30	L	78
Gyrus supramarginalis	40	−57	−52	25	3.12	L	180
**Lobus parietalis**							
Precuneus	31	−9	−49	34	3.02	L	67
**Post-Acute patients with paranoid schizophrenia**							
**Lobus frontalis**							
Gyrus frontalis inferior	46	−45	29	16	2.98	L	125
**Lobus limbicus**							
Gyrus parahippocampalis	28	3	−28	−41	3.05	R	67
**Lobus temporalis**							
Gyrus temporalis superior	22	48	−22	−5	3.04	R	52
Gyrus temporalis superior	39	42	−49	7	2.96	R	353
Gyrus temporalis inferior	20	−57	−58	−14	3.32	L	658
**Lobus parietalis**							
Gyrus supramarginalis	40	57	−37	28	3.82	R	3991
Gyrus postcentralis	1	−64	−22	40	3.28	L	225
Lobulus parietalis inferior	40	−51	−37	31	3.65	L	2847
**Lobus occipitalis**							
Gyrus occipitalis medialis	37	51	−64	−11	3.17	R	1076
Gyrus occipitalis medialis	19	−36	−67	10	3.35	L	96
Gyrus occipitalis inferior	18	40	−85	−8	3.28	R	253
Gyrus occipitalis inferior	18	−42	−91	−2	3.08	L	79
Gyrus fusiformis	18	24	−95	−17	3.18	R	334
Gyrus fusiformis	18	−30	−98	−14	3.41	L	606
**Healthy Participants**							
**Lobus frontalis**							
Gyrus frontalis superior	8	−3	44	55	2.97	L	98
Gyrus frontalis superior	6	6	−1	71	3.10	R	110
Gyrus frontalis medialis	46	−51	32	25	3.33	L	126
Gyrus frontalis medialis	6	−54	8	40	3.31	L	121
Gyrus frontalis inferior	47	−33	26	−17	2.94	L	50
Gyrus frontalis inferior	45	60	23	10	3.28	R	480
Gyrus frontalis inferior	9	51	17	25	3.50	R	1572
**Lobus limbicus**							
Uncus	36	15	−13	−32	3.44	R	100
Gyrus parahippocampalis	35	−9	−28	−35	3.02	L	77
Cingulum posterior	29	9	−46	19	3.67	R	466
**Lobus temporalis**							
Gyrus temporalis superior	38	−36	20	−26	3.25	L	213
Gyrus temporalis superior	22	−60	−40	22	3.27	L	886
Gyrus temporalis medialis	21	48	−1	−17	3.33	R	379
Gyrus temporalis medialis	39	48	−58	10	3.56	R	9942
**Lobus occipitalis**							
Gyrus occipitalis medialis	37	−45	−67	4	3.27	L	881
Gyrus occipitalis inferior	18	−39	−91	−14	2.96	L	54
***Random > Theory of Mind***							
**Acute patients with paranoid schizophrenia**							
**Lobus occipitalis**							
Gyrus lingualis	18	−18	−79	−11	−3.49	L	513
Cuneus	17	6	−88	7	−3.13	R	116
**Post−acute patients with paranoid schizophrenia**							
**Lobus frontalis**							
Gyrus frontalis medialis	9	21	41	13	−3.37	L	279
**Lobus limbicus**							
Gyrus cingularis	32	−21	14	31	−3.61	L	611
Gyrus cingularis	24	−24	−19	40	−3.37	L	319
**Healthy participants**							
**Lobus limbicus**							
Gyrus cingularis	24	21	−13	43	−3.14	R	210
**Lobus parietalis**							
Lobulus parietalis superior	7	−27	−58	65	−3.21	L	252

BA = Brodmann area, Z = Z-Score (peak of brain region), L = left, R = right, voxel = size of activation ; level of significance: *p* < 0.01.

**Table 5:** Neuronal activations responsive to the ToM condition (compared to the random condition) in acute patients and post-acute patients with paranoid schizophrenia compared to healthy controls and vice versa.

Talairach Coordinates								
Brain Region		BA	x	y	z	Z	r/l	Voxel
***Theory of Mind > Random***								
**Acute Patients with Paranoid Schizophrenia**								
**Lobus Frontalis**								
Gyrus frontalis medialis		9	−21	44	13	4.92	L	548
Gyrus frontalis medialis		6	21	−4	37	3.49	R	73
**Lobus limbicus**								
Gyrus cingularis		31	18	−19	40	3.42	R	127
Gyrus parahippocampalis		30	27	−46	4	3.48	R	80
**Healthy participants**								
**Lobus frontalis**								
Gyrus frontalis superior		6	6	38	58	−2.90	R	56
Gyrus frontalis medialis		46	−54	29	25	−3.91	L	353
Gyrus frontalis medialis		8	−48	26	40	−4.23	L	446
Gyrus frontalis medialis		46	60	23	25	−3.25	R	174
Gyrus frontalis inferior		45	64	20	16	−3.14	R	134
Gyrus precentralis		6	−61	8	37	−3.26	L	125
**Lobus limbicus**								
Uncus		28	18	−15	−32	−3.55	R	124
Gyrus parahippocampalis		37	12	−19	−8	−3.44	R	169
Cingulum posterior		29	0	−49	10	−3.19	L	62
**Sub−lobar**								
Nucleus lentiformis	Putamen		30	5	4	−3.69	R	169
Nucleus lentiformis	Putamen		−21	−16	4	−3.91	L	270
**Lobus temporalis**								
Gyrus temporalis medialis		39	48	−58	10	−3.43	R	496
**Post-acute patients with paranoid schizophrenia**								
**Lobus parietalis**								
Lobulus parietalis inferior		40	58	−43	43	3.83	R	2534
**Healthy participants**								
**Lobus frontalis**								
Gyrus frontalis medialis		11	−42	41	−11	−4.70	L	237
**Lobus limbicus**								
Gyrus cingularis		24	−18	11	28	−3.83	L	477
Gyrus cingularis		31	−24	−22	37	−3.51	L	80
Gyrus cingularis		23	−3	−22	−23	−3.24	L	224
**Sub-lobar**								
Nucleus caudatus	Caput nuclei caudati		24	−31	13	−3.10	R	60
Nucleus lentiformis		--	−18	−7	7	−3.61	L	138

BA = Brodmann area, Z = Z-Score (peak of brain region), L = left, R = right, voxel = size of activation ; level of significance: *p* < 0.01.

## Data Availability

This study, based in part on the unpublished doctoral thesis of C.F., can be downloaded at https://edoc.ub.uni-muenchen.de/13452/ (accessed on 24 January 2018).

## Appendix A

**Table A1:** Scoring criteria and examples for verbal descriptions of animations.

**Appropriateness**	**Accuracy of the Description**
0	No answer, little information available to evaluate
1	Incorrect, bizarre answer, reference to the wrong type of interaction between triangles
2	Partially correct answer, reference to correct type of interaction but confused overall description
3	Appropriate, clear answer
**Intentionality**	**Type of Description**
0	Nondeliberate action (moving around, rotating)
1	Deliberate action with no other (ice-skating)
2	Deliberate action with another (fighting, following)
3	Deliberate action in response to another’s action (chasing, guarding)
4	Deliberate action in response to another’s mental state (mocking, arguing)
5	Deliberate action with goal of affecting another’s mental state (surprising)
**Humanization**	**Representation of the Triangles**
0	Triangles with no humanized description
1	Partially humanized triangles, with a goal
2	Humanized triangles, with thought and feelings
3	Humans
